# Improved antibacterial activity of a sustained-release biocompatible nanofilm for treating *Pseudomonas aeruginosa* wound infection *in vitro* and *in vivo*

**DOI:** 10.3389/fmicb.2025.1548106

**Published:** 2025-04-09

**Authors:** Feifei Lu, Guoxiu Lu, Zhiguo Wang, Wenzhua Wei, Jingjing Chen, Hailin Zheng, Xuesong Liu, Yan Ye, Shuling Liu, Yuxiang Lin, Yuxin Liu, Zhanhua Bi, Quanming Zou, Guoxu Zhang, Hongwu Sun, Yanan Tong

**Affiliations:** ^1^College of Medicine and Biological Information Engineering, Northeastern University, Shenyang, Liaoning, China; ^2^Department of Nuclear Medicine, General Hospital of Northern Theater Command, Shenyang, China; ^3^National Engineering Research Centre of Immunological Products, Department of Microbiology and Biochemical Pharmacy, College of Pharmacy, Third Military Medical University, Chongqing, China

**Keywords:** multidrug-resistant bacterial, *Pseudomonas aeruginosa*, nanofilm, antibacterial effect, wound heal

## Abstract

**Objectives:**

The primary goal of this research was to design a useful and biocompatible nanofilm system (CNF) encapsulating chlorhexidine acetate (CHX) for wounds that is endowed with antibacterial and anti-inflammatory activities and promotes wound healing.

**Methods:**

The nanofilm system was developed on the basis of the successful preparation of its nanoemulsion and PVA-CS film system and then important properties of the nanofilm system, including its morphological and physicochemical characteristics, stability and safety, its antimicrobial efficacy against *P. aeruginosa* was also evaluated *in vitro* and *in vivo*. The antibacterial effect, wound healing effect and inflammatory factor change *in vivo* were evaluated.

**Results:**

These results of this nanofilm system revealed a good particle size (59.27 nm) and stable zeta potential (−15.2 mV) that are suitable for wound healing applications. Additionally, it was stable, exhibited long-term stability (24 months) and sustained release in simulated wound fluid. Results showed that this nanofilm does not induce dose-related toxic effects and displays a better antibacterial effect that occurs more quickly, two times greater than that of CHX *in vitro*. This safe nanofilm enhances antibacterial activity against *P. aeruginosa* for 14 days, modulates the immune response, and accelerates skin wound healing *in vivo*.

**Conclusions:**

These insights into multifunctional nanofilm designs for improved antibacterial effects and sustained release suggest promising clinical applications.

## 1 Introduction

Wound infections caused by pathogenic bacteria pose a major health risk due to complications and delayed healing (Zhou et al., 2024). The delayed healing of these wounds may lead to severe sequelae, including sepsis and death (Ouyang et al., 2022), highlighting the need for effective management. The incidence rate of wound site infection is about 30%. Even if there is sufficient antibiotic use and support treatment, the mortality rate is still 40–50%. Especially when multiple drug resistant bacterial infection occurs, the mortality rate will be even higher. *Pseudomonas aeruginosa* (*P. aeruginosa*, PA), a major opportunistic pathogen responsible for chronic wound infections, poses a significant threat because of its intrinsic antibiotic resistance and ability to cause multiorgan failure. The existence of drug-resistant strains exacerbates this situation, as they delay healing and increase the risk of mortality. The pervasive use of antibiotics has fueled healing resistance, limiting therapeutic options and increasing costs (Valappil et al., 2024; Xie et al., 2024; Soleymani et al., 2024).

The traditional therapeutic approach for bacterial wound infections involves the use of antibiotics, which, unfortunately, has led to the emergence of drug-resistant bacterial strains and compromised the effectiveness of treatments aimed at combating wound infections (Zulfiqar et al., 2024). In view of the infection of PA, the development of antibiotics is far behind the rate of bacterial resistance. Currently, antibiotics that can be effectively applied in clinical are limited. Cephalosporins, carbapenems, fluoroquinolones, aminoglycosides and polypeptides are the main antibiotics for PA infection, But monitoring data show that the resistance of chloromyces to these antibiotics is rising, and it is likely to face the situation of no drug availability. Standard antibacterial dressings, though commonly used, have been found to manage infections inadequately and foster the development of antibacterial resistance inadvertently (Rezaie et al., 2024). Therefore, the development of novel drugs or formulations to control infections caused by multidrug-resistant bacteria is urgently needed.

In light of the mounting challenge posed by multidrug resistance against antibiotics, strategies that do not rely solely on antibiotics have emerged as promising avenues for anti-infective therapy (He et al., 2024). Research has conclusively shown that chlorhexidine (CHX), which is a broad-spectrum nonantibiotic agent routinely employed for cleansing a diverse array of surgical instruments and devices, is a robust disinfectant (Denkel et al., 2022). The antimicrobial attributes of CHX have garnered extensive attention for prolonged antibacterial applications, positioning it as a viable candidate for novel topical formulations (Hemmingsen et al., 2021; Shamshad et al., 2023). Compared with that to target-specific compounds, resistance to this phytocompound is lower because of its rapid and broad bactericidal activity. Its synergy with chitosan enhances bacterial eradication. However, the low solubility and instability of CHX hinder their sustained delivery for clinical use (Wen et al., 2021). Consequently, ongoing research has focused on overcoming these challenges to expand the applications of CHX.

Nanotechnology has emerged as a promising field in medicine (nanomedicine), exploiting the unique physical and chemical attributes of nanoscale structures for potent diagnostic and therapeutic applications. Nanoemulsions, as colloidal delivery systems, demonstrate remarkable loading capacities and the ability to promote the stability of bioactive compounds, garnering significant interest in nano-medicine research. Nanoemulsions, formed by emulsifying aqueous and oily phases, range in size from 1 to 00 nm. Their appearance varies depending on the droplet size, and they offer advantages over other nano-carriers, including easy preparation, enhanced stability, and improved skin bio-availability (Chong et al., 2024). However, nanoemulsions have several limitations, including a tendency to flow easily and a relatively brief residence period. A variety of wound dressings have been commercialized, incorporating alginates, hydrogels, and hydrocolloids as their primary forms. These dressings can be applied as thin, adhesive layers or sheets suitable for both dry and wet wound surfaces, ensuring a secure fit for patients. Notably, hydrogels stand out as highly suitable candidates for wound dressing applications owing to their ability to mimic the extracellular matrix for cell adhesion, maintain optimal moisture levels at the wound site, and facilitate gentle removal to mitigate the risk of secondary damage (Chamchangi et al., 2024). Optimal wound dressings require a range of attributes, including the ability to sequester exudate, prevent infections, maintain moisture content, facilitate atmosphere exchange, and be biocompatible and biodegradable. Their adhesion is not excessive, which means easy replacement and accelerated angiogenesis and tissue regeneration (Arezomand et al., 2024). Among popular materials are films, foams, sponges, hydrogels, nanofiber and nanofilm systems. Nanofilms are ideal for this situation because of the following advantages: enhanced therapeutic efficacy and wound-healing activity, reduced side effects, and minimized administration frequency. Owing to their unique structure, nanofilms provide a barrier against infection while facilitating the transport of gases and liquids because of their high porosity and small pores. To promote wound recovery and develop ideal wound dressings with multiple functions, including antibacterial and anti-inflammatory effects, polymers belonging to the cationic polysaccharide family, notably chitosan and its derivatives, have emerged as promising candidates for wound healing endeavors. The exceptional absorbency, capacity to form films, and inherent antimicrobial and antioxidant properties of these materials confer significant potential. These polymers can enhance hemostasis, combat microorganisms, and regulate inflammation, making them more effective in wound care. Previous studies have reported that chitosan-based nanocomposite films are thin, soft, and transparent, which facilitates air exchange in the wound microenvironment (Arezomand et al., 2024; Wang et al., 2022). There have been no reports on the use of safe and stable acetic acid/CHX nanofilm systems, which are based on PVA/CS films and nanoemulsion systems, with antibacterial and antioxidant properties.

**Figure 1:**
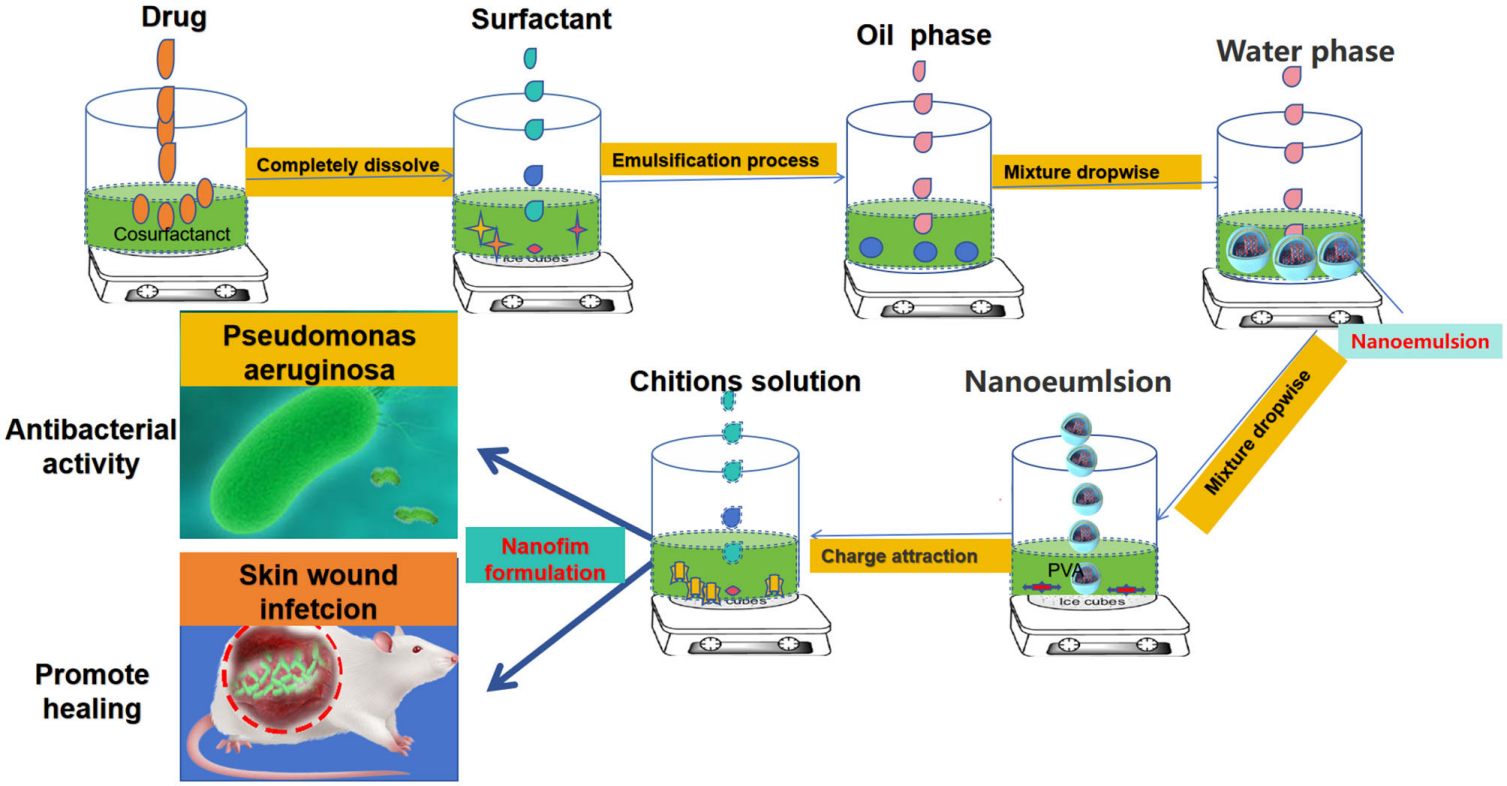
Design of a novel nanofilm system that promotes antibacterial activity and accelerates wound healing.

The primary goal of this research was to design a useful dressing for wounds that is endowed with antibacterial and anti-inflammatory activities and promotes wound healing. As shown in Figure 1, the nanofilm system was successfully developed. After evaluating the important properties of the nanofilm system, including its morphological and physicochemical characteristics, stability and safety, its antimicrobial efficacy against *P. aeruginosa* was also evaluated *in vitro* and *in vivo*. The antibacterial effect, wound healing effect and inflammatory factor change *in vivo* were evaluated. The results showed that CNF had stronger antibacterial and healing effects than CHX, and stronger effect on down regulation of Pro inflammatory factor and up regulation of anti-inflammatory factor. These results indicate that this system is a robust and ideal formulation for treating wounds infected with *P. aeruginosa* and will provide a solid theoretical basis and technical support for the research of the delivery system of wound antimicrobial agents.

## 2 Materials and methods

### 2.1 Bacteria, animals, and ethics statement

*P. aeruginosa* PAO1 (ATCC 27853) was obtained from the American Type Culture Collection (ATCC; USA) and stored at −80°C. The bacteria were cultured in LB broth (AOBOX Biotechnology, China) and LB nutrient agar (AOBOX Biotechnology, China) for 16 h of incubation at 37°C. HFK Bioscience Co., Ltd., Beijing, provided specific pathogen-free (SPF) BALB/c mice (6–8 weeks old, female). The mice were kept in an SPF laboratory (Third Military Medical University, China) at 24°C and a relative humidity of 50% with half-day light and dark cycles. The animal tests were authorized by the Animal Ethical and Experimental Committee Center (Third Military Medical University, No. AMUWEC2020973).

### 2.2 Design and preparation of the novel nanofilm system

The preparation of the nanoemulsion and film-forming systems followed previously described instructions (Arezomand et al., 2024; Yang et al., 2018; Cai et al., 2022). This 1% (w/v) nanoemulsion was manufactured using the phase inversion method via the addition of chlorhexidine acetate (CHX; Jinzhou Jiutai Pharmaceutical Co., Ltd., China), Tween 80 (Shanghai Sinopharm Group, China), propylene glycol (Shanghai Sinopharm Group, China), and isopropyl myristate (IPM; BASF, Germany). Then, PVA (polymerization degree: 600–800; viscosity: 4–6 mPa·s; degree of alcoholysis: 88 ± 2%; Meijia Meng Technology Limited Company of Chongqing, China) was weighed and added in accordance with a 10% (w/v) ratio, the mixture was stirred intermittently, and a small amount was added to cold water. After all the components were added, the mixture was stirred at 200 rpm for more than 6 h until the nanoemulsion fully swelled. Chitosan (CS; w/v: 1%; acetylation: 85%; viscosity: 20–200 mPa·s; molecular mass: 50–190 kDa; Sigma–Aldrich Company, USA) was added to a 1% nanoemulsion and 5% PVA, and the mixture was mixed for 10 min at 200 rpm. The additives were subsequently gradually introduced under gently stirring until a homogenous film-forming solution was obtained. Using the above techniques, a blank nanofilm system (BNF) was created without drug addition.

### 2.3 Film formation and characteristic observation of the nanofilm system

The transparency and color of the 5 mg/mL CHX-containing nanofilm (CNF) were observed under normal light with a white background. In brief, 50 μL of solution was placed on a glass slide to evaluate their film-forming ability. The glass slide was then dried at 37 ± 0.5°C in a DGF20022B hot air oven (Chongqing, China). As described in a prior publication (Yang et al., 2018), the stability of the solution over time and the thickness of the film were visually examined. After the samples were diluted to a concentration of 1% in water (El-Abeid et al., 2024), the hydrodynamic size distribution, PdI, and ζ potential (zeta potential) of the samples were measured with a Nano ZS instrument (Malvern Lit. Co., UK). Furthermore, after the samples were diluted 100-fold in water, the morphology of the nanofilm was examined via an FEI TECNAI10 transmission electron microscope (Philips Electron Optics, Holland) running at a voltage of 120 kV. A scanning electron microscope (S-3400N, Hitachi) was also used to analyze the samples.

### 2.4 Physical and stability characteristics of the nanofilm system

The physical and stability characteristics of the nanofilm system were evaluated via previously reported methods (Luo et al., 2023; Sang et al., 2024). Thermogravimetry (TG) and differential scanning calorimetry (DSC) were performed in an inert atmosphere of nitrogen at a rate of 10°C/min with TA instruments (Q600, New Castle, USA). Following the generation of KBr disks, the distinctive absorption peaks were examined via Fourier transform infrared (FTIR, Lambda 950 spectrometer, PerkinElmer, Boston, USA) spectroscopy, with a wavelength range of 400–4,000 cm^−1^.

Through six cycles of centrifugation experiments (3,000 × g, 10 min) with alternating temperature changes from 4 to 25°C, instability phenomena such as precipitation and turbidity were observed. The samples were diluted 1:100 with ultrapure water at room temperature (25 ± 5°C) and stored for 0, 6, 12, and 24 months. The size, PdI, and ζ potential of the samples were immediately determined at 25°C via a size and zeta analyzer (Zetasizer Nano ZS90, Malvern, UK) at 633 nm. The scattering intensity was measured at a scattering angle of 173° relative to the source using a cascade of photodiode detectors at 25°C. Intensity autocorrelation functions were analyzed using general purpose algorithm software (Malvern Zetasizer) to determine the distribution of the translational Z-averaged diffusion coeffcients of the particles.

### 2.5 *In vitro* release dynamics of the nanofilm system

To determine the release dynamics of this nanofilm system, drug release was determined via HPLC (Waters^®^, E2695; Waters, MA, USA) (Li et al., 2015; Chen et al., 2024). *In vitro* release studies were performed in simulated wound fluid (SWF) (Yang et al., 2018) at pH 7.4 and 37°C. The test bags were soaked in release medium at a stirring rate of 100 rpm at room temperature. Approximately 2 mL CNF and CHX (drug concentration: 5 mg/mL) was placed in a 10,000 g/mol molecular weight cutoff dialysis bags (Sangon Biotech Limited Company, Shanghai, China). The same volume of fresh release medium was added to maintain the same volume. The sample solution was centrifuged at 10,000 × g for 10 min and the supernatant liquid was measured using the HPLC methods described above.

### 2.6 *In vitro* antibacterial effects of the nanofilm system

The microdilution method was used to measure the minimum inhibitory concentration (MIC), as previously reported (Yassin et al., 2023). Briefly, each well of a 96-well plate was filled with a PAO1 bacterial mixture (10^6^ CFU/mL, CFU is short for the colony-forming unit) with a series of CNF and CHX concentrations (final concentrations: 50, 25, 12.5, 10, 6.25, 5, and 3.125 μg/mL). Following 16 h of incubation at 37°C, the absorbance at 600 nm was assessed via a Bio-Rad 6.0 reader (Bio-Rad, USA). The control groups included PAO1 positive control, BNF blank control and LB negative control. The MIC value is considered the lowest sample concentration at which no visible bacterial (OD_600nm_ < 0.05; Cai et al., 2022) growth can be observed (Ly et al., 2024).

On the basis of the CNF and CHX MICs (2 × MIC−0.5 × MIC) reported in our earlier study (Salih et al., 2024), the concentrations used for the time–kill test were selected. CHX and CNF were added to the bacterial suspensions (1 × 10^6^ CFU/mL) at three different concentrations: 3.12, 6.25, and 12.5 μg/mL. The suspensions were incubated for 1, 5, 15, 30, 45, 60, 120, 240, 480, or 640 min. At each time point, 5 μL of each suspension was sampled; a series of 10-fold dilutions of the samples were made and added to LB plates, which were then incubated for 16–20 h at 37°C. The number of bacterial colonies was determined with an automatic colony counter (Shineso S&T Limited Company, Hangzhou, Zhejiang, China; Yang et al., 2018). BNF was concurrently diluted to the same degree as the maximum concentration of CNF for use as a blank control.

### 2.7 *In vitro* cytotoxicity assessment of the nanofilm system

To ascertain the cytotoxicity of this novel system (Narayanan et al., 2024), a 96-well microtiter plate containing L929 cells was used to conduct a 3-(4,5-dimethylthiazol-2-yl)-2,5-diphenyltetrazolium bromide (MTT) test. The L929 cells were cultivated at a density of 1 × 10^4^ cells/well in DMEM with 1% antibiotic and 10% fetal bovine serum (FBS). The samples were then incubated for 1 day at 37°C in a CO_2_ incubator. Next, 100 μL of various concentrations (15.65–1,000 μg/mL) of CNF was added, and the combination was incubated for 24 h under the previously described conditions. For the blank control, the blank self-nanoemulsion (BNF) was diluted to prepare the solution with the highest concentration. Next, 100 μL of MTT was added to the cells. Later, the medium was fully aspirated, and a microplate reader set to 570 nm was used to assay crystal formation.

### 2.8 *In vivo* safety evaluation of the nanofilm system

The general guidelines from ISO10993-5 concerning the biological evaluation of medical devices were followed (Ramirez-Labrada et al., 2024). On the day before administration, the dorsal skins of the mice were shaved and disinfected with 75% ethanol. A skin incision of ~1.0 × 1.0 cm^2^ was made on the dorsal surface and subcutaneous tissue dissection was performed under aseptic conditions. Full-thickness wounds were generated by blunt dissection of the skin. The mice were then divided into 3 groups (*n* = 5): the 5 mg/mL CHX, 5 mg/mL CNF and PBS control (without drug treatment) groups. All the mice were topically administered 100 μL of each solution to the local skin region for 30 s once daily, with treatment for 14 days. The mice were anesthetized with pentobarbital before being sacrificed via CO_2_ inhalation. For histological analysis, the heart, liver, spleen, lungs, kidneys, and skin were excised, followed by fixation in 4% paraformaldehyde and paraffin embedding. Hematoxylin and eosin (H&E) staining was applied to sections from these organs. *In vivo* tissue safety was analyzed via an optical microscope (Olympus BX53, Olympus, Japan).

### 2.9 Preparation of the bacterial wound infection animal model

A total of 24 mice were split into four groups (*n* = 6) at random: the PA infection, 5 mg/mL CHX, and 5 mg/mL CNF groups, with BNF acting as a control. The mice were intraperitoneally injected with pentobarbital for anesthesia. Then, their dorsal hair was removed, and a full-thickness incision (1.0 × 1.0 cm^2^) was made on their backs. All the mice were infected with 200 μL of an OD_600nm_ = 2.0 bacterial suspension (2 × 10^9^ CFU/mL) for 30 s. Ten minutes later, 100 μL of solution was topically applied to the skin incision area of each mouse for 30 s.

### 2.10 Cytokine levels in *P. aeruginosa*-infected wounds

The mice were euthanized at 12 and 24 h after administration (*n* = 3); then, their eyeballs were immediately removed, and blood was drawn and stored at 4°C for 24 h. Next, the serum was separated via centrifugation for 5 min at 8,000 rpm. The secretion levels of the cytokines interleukin 1β (IL-1β), tumor necrosis factor α (TNF-α), interleukin 6 (IL-6), and interleukin 10 (IL-10) in the serum were determined via cytokine assay kits (Dakewei Biotech Co., Ltd., Beijing, China).

### 2.11 Effectiveness of wound healing on the basis of CFU counts

On day 4, bacterial counts were determined as follows. The wound surface of each mouse was swabbed 10 times with sterile cotton swabs. The swab was then placed into a sterile 1.5-mL physiological saline centrifuge tube and mixed 10 times to release bacteria into the liquid. Each sample was then serially diluted 5 times with sterile physiological saline solution by adding 2 μL of the solution to LB nutrient agar, which was incubated for 16–20 h. After incubation, the number of CFUs on the plates was counted to calculate the actual number of colonies.

### 2.12 Healing ratio and pathological changes in the wound area

The wound area in each group was recorded and analyzed via ImageJ software on days 1, 4, 7, 10, and 14. On day 7, one mouse from each group was randomly selected. After the mice were subjected to anesthesia, wound tissue was collected and preserved in 10% formaldehyde for a full day (24 h). On the 17th day of the *in vivo* trial, the tissue was sealed with paraffin. Tissue sections (5 μm) were prepared using a microtome after the material had been embedded in paraffin. The sample was subsequently placed onto a glass slide, and H&E staining was used to evaluate the shape of the skin and the synthesis of collagen. The samples were analyzed and examined via an Olympus BX53 optical microscope (Japan). Digital images of the wounds were captured, and the wound areas (length × width) were computed.

### 2.13 Statistical analysis

Statistical analyses of all the data were conducted via GraphPad Prism software (v. 8.0.1, GraphPad, USA). The data are shown as the means ± SDs or means ± SEMs. Comparisons between two groups were made via the unpaired *t*-test, and comparisons among more than two groups were made via univariate analysis of variance. The Newman–Keuls test was used to examine group differences. Statistical significance is indicated by asterisks (^*^, ^**^, or ^***^) for *P*-values < 0.05, 0.01, and 0.001, respectively.

## 3 Results

### 3.1 Design and preparation of the novel nanofilm system

In this study, we employed CHX for the development of a novel nanofilm. Using it as the active drug, a novel nanofilm system (CNF) was developed on the basis of the successful preparation of its nanoemulsion and PVA-CS film system. This system not only endows the nanofilm with antibacterial properties but also improves its wound healing activity (Figure 1).

### 3.2 Appearance and film-forming ability of the novel nanofilm system

**Figure 2:**
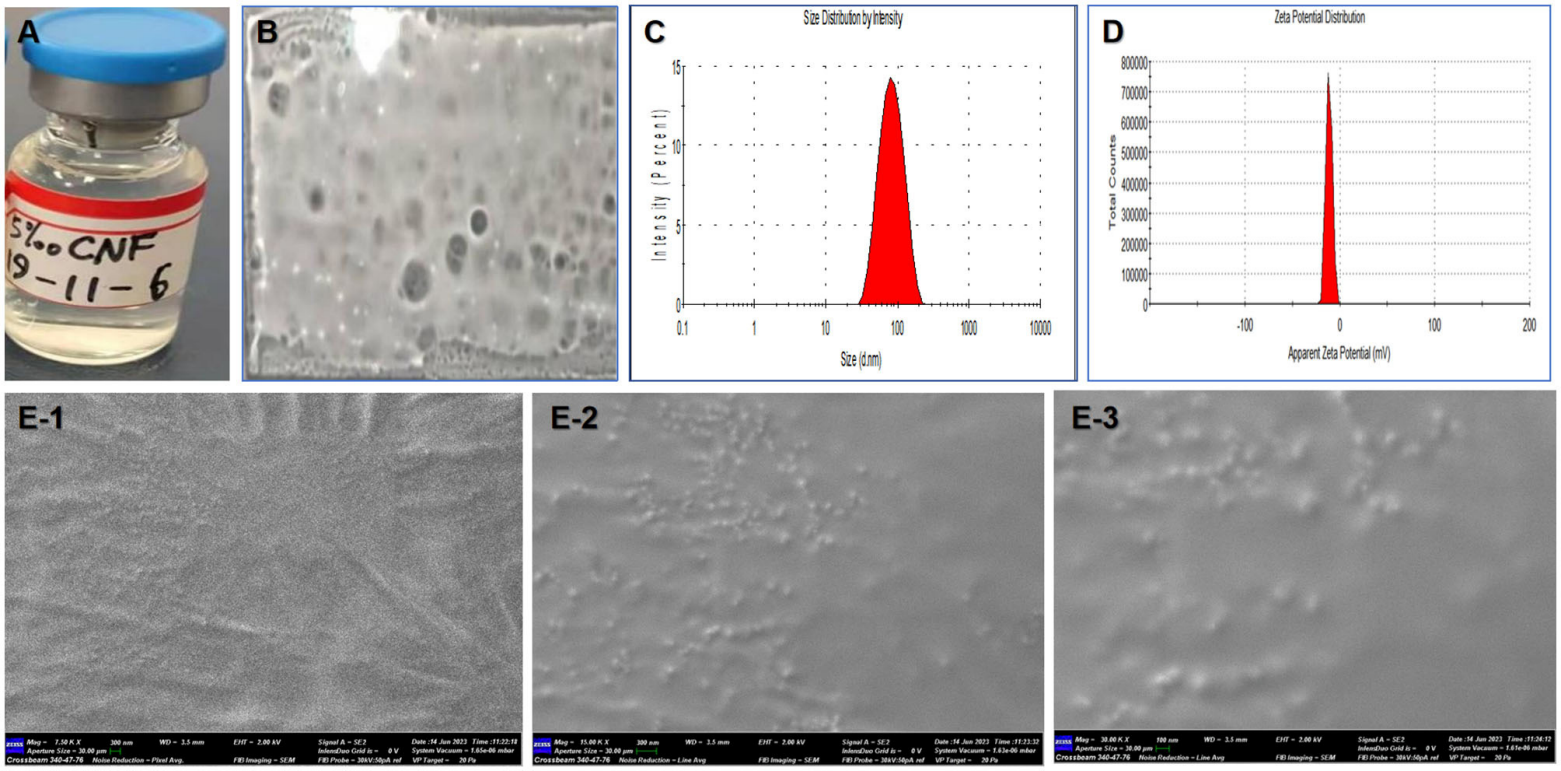
Film-forming and physical properties of the nanofilm system. **(A)** Appearance. **(B)** Film-forming ability. **(C)** Size distribution. **(D)** Zeta potential distribution. **(E)** Surface morphology observation by SEM (1–3 correspond to 7.5, 15, and 30 k × magnification, respectively).

The appearance of the nanofilm system (CNF) is shown in Figure 2A. A small amount of precipitate was present in the CHX (0.5%, 5 mg/mL) solution, whereas the CNF (0.5%, 5 mg/mL) was clarified. Since the emulsion and nanofilm system materials themselves are not completely colorless, the finished nanofilm system has a slight coloration. Images of the films formed after coating are shown in Figure 2B. The formed films were free from defects and had excellent film-forming characteristics. Further study revealed that in an oven at 37°C, the film formation time of the preparation was 2 min, and the film could be maintained in the oven for 5 h.

### 3.3 Size and zeta potential of the novel nanofilm system

The average particle size and potential were determined by a Nano ZS instrument, and the results are shown in Figures 2C, D. The particle size of the 5 mg/mL CNF was ~59.71 nm (PdI = 0.266), and a single main peak at 43.2 nm was observed. In addition, the ζ potential and pH were −15.2 mV and 6.30, respectively, and the electrical conductivity was 0.131 mS/cm. The electrophoretic mobility rate was −1.211 μm/v.

### 3.4 Surface morphology of the novel nanofilm system

**Figure 3:**
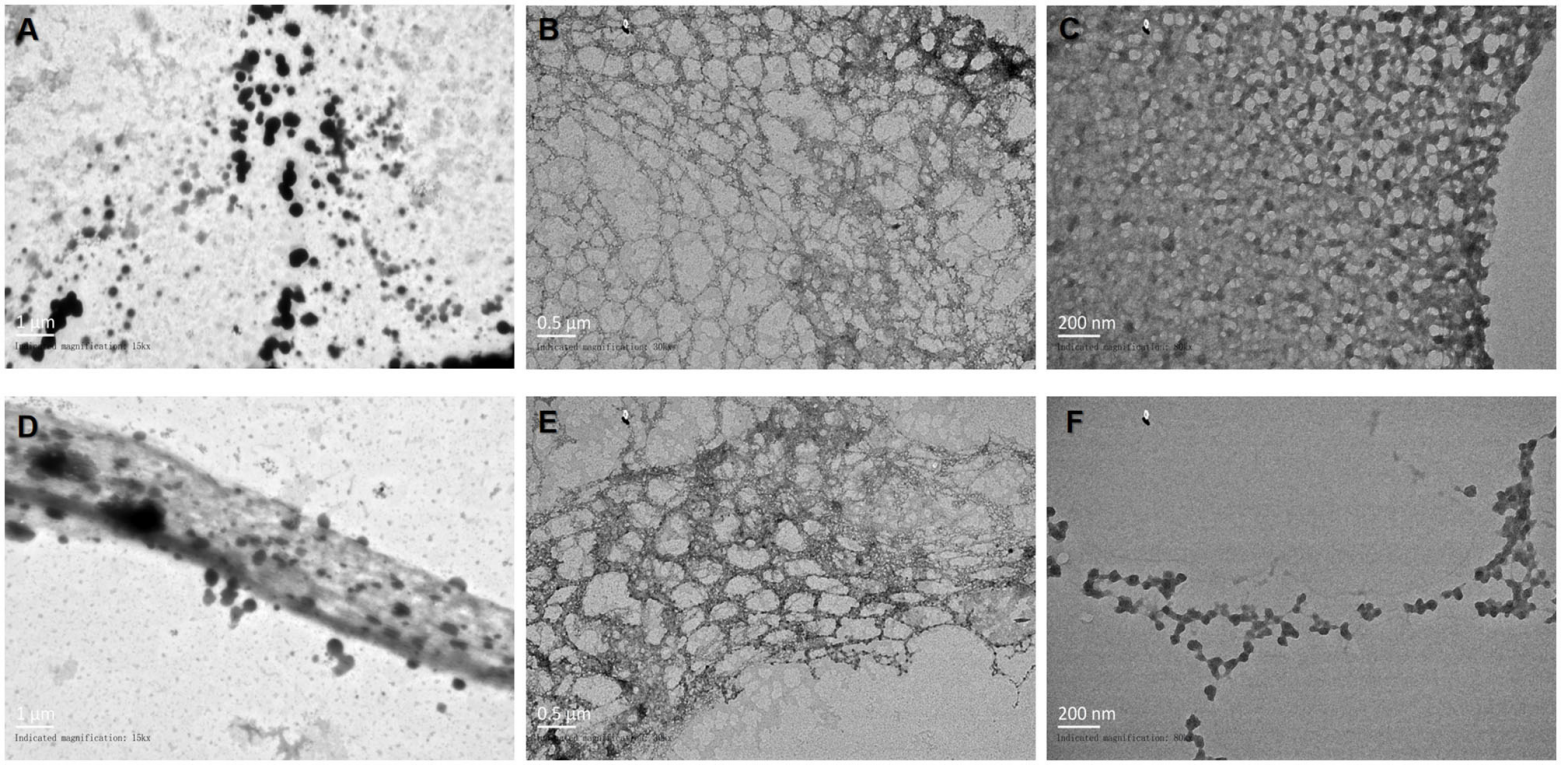
Observation of the morphology of the novel nanofilm system by TEM. **(A, D)** 15 k × magnification. **(B, E)** 30 k × magnification. **(C, F)** 60 k × magnification.

Scanning electron microscopy (SEM) images of the CNF are shown in Figures 2E-1–E-3. The SEM micrographs show that the nanoemulsion particles are clustered together and surrounded by a polymer sheath. The average pore diameter of the macroporous structure is 1–100 nm. Porosity is a key factor in the ability of a material to support the healing process, as it makes the delivery of nutrients easier by allowing vascularization. Many ridges within the nanoemulsion particles and film material can be observed at a magnification of 7.5 k × (Figure 2E-1). More and clearer nanoemulsion particles appeared at magnifications of 15 k × (Figure 2E-2) and 30 k × (Figure 2E-3). Therefore, the size range of the nanofilm is suitable for further delivery and targeting research.

### 3.5 Morphological characterization of the novel nanofilm system

The results of transmission electron microscopy (TEM) morphological characterization of the novel nanofilm are shown in Figures 3A–F. The CNF was diluted 100-fold with water and magnified by TEM 15 k × . The loaded drug appeared as black particles (Figures 3A, D). At a magnification of 30 k × , a fibrous structure was observed (Figures 3B, E), and further magnification at 60 k × revealed that this CNF had a reticular structure (Figures 3C, F). Therefore, these particles were well dispersed and evenly distributed in the nanofilm, which demonstrates their consistent morphological distribution.

### 3.6 Structural characterization of the novel nanofilm system

**Figure 4:**
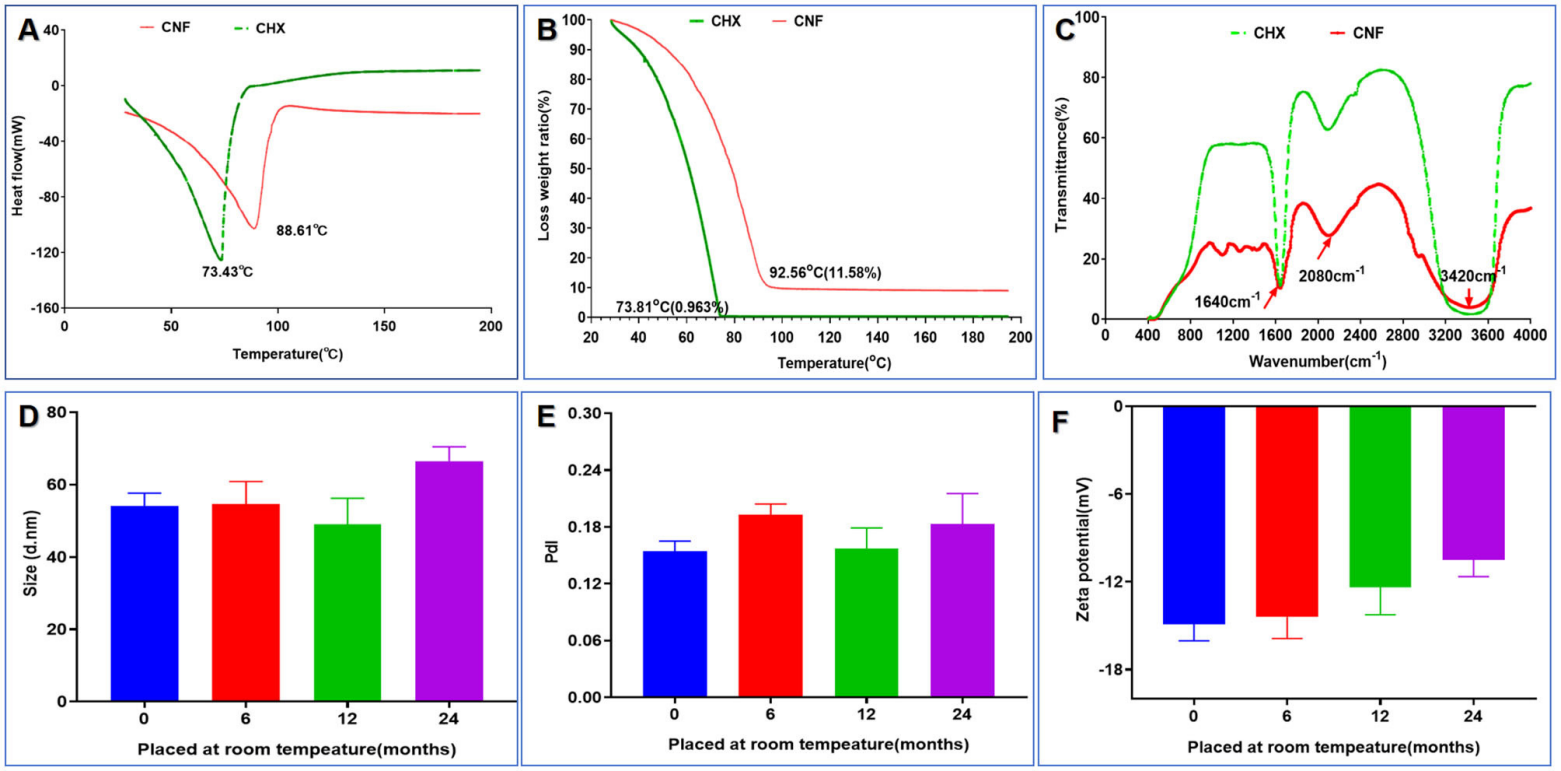
Structural and stability characterization of the novel nanofilm system. **(A)** DSC. **(B)** TG. **(C)** FITR. **(D)** Size change (*n* = 3). **(E)** PdI change (*n* = 3). **(F)** Zeta potential change (*n* = 3). *P < 0.05; **P < 0.01; ***P < 0.001, ANOVA.

Digital scanning calorimetry (DSC) isometric techniques measure the heat flow associated with the thermal and phase transitions of materials and are routinely used in nanoscience to calculate the thermodynamic properties of nanomaterials and biomolecules. DSC revealed that there was a distinct peak at 74.43°C for the CHX aqueous solution in the thermogram, whereas there was no peak for the CNF (Figure 4A). In addition, a peak at 88.61°C appeared for the CNF, indicating that drug encapsulation in the nanofilm formulation resulted in a peak shift on DSC. This characteristic peak of the CNF was not observed for the film loaded with CHX, demonstrating that the drug was molecular well dispersed. Additionally, solid-state characterization of this nanofilm system was performed via thermogravimetric (TG) analyses. The characterizations include the determination of drying loss, thermal stability and phase transition temperatures to determine whether water is bound or unbound. TG analysis revealed a peak for the highest rate of weight loss. We found that the weight loss of both CHX and CNF started at ~20°C, and the temperatures with the maximum rates of weight loss were ~73.81 and 92.56°C (Figure 4B), respectively. These results indicate that this nanofilm system was successfully loaded with the drug. In addition, FTIR spectroscopy revealed three main characteristic peaks at 3,420, 2,080, and 1,640 nm, corresponding to O–H stretching, C–H stretching and amide I, which were found in the CNF and CHX (Figure 4C). These results confirmed the success of CHX loading into the nanofilm system.

### 3.7 Stability of the nanofilm system

Through six cycles of centrifugation (3,000 × g, 10 min) with alternating temperatures from 4 to 25°C, no unstable phenomena, such as flocculation, delamination, drug precipitation, or sedimentation, occurred before or after centrifugation of the nanofilm system, indicating that the prepared nanofilm system was stable. The important physical properties of the nanofilm system, such as the particle size, PdI, and zeta potential, were measured at room temperature at 0, 6, 12, and 24 months, as shown in Figures 4D–F. The particle size of the system ranged from 48.99 to 66.51 nm, as shown in Figure 4D. The PdI values fluctuated very little, ranging from 0.24 to 0.14 (Figure 4E). Finally, as shown in Figure 4F, the ζ potential of this system varied from −14.9 to −10.49 mV, suggesting high stability. These results indicate that there was no significant change after 24 months of storage at room temperature (all *P-*values > 0.05).

### 3.8 *In vitro* release dynamics of the nanofilm system

**Figure 5:**
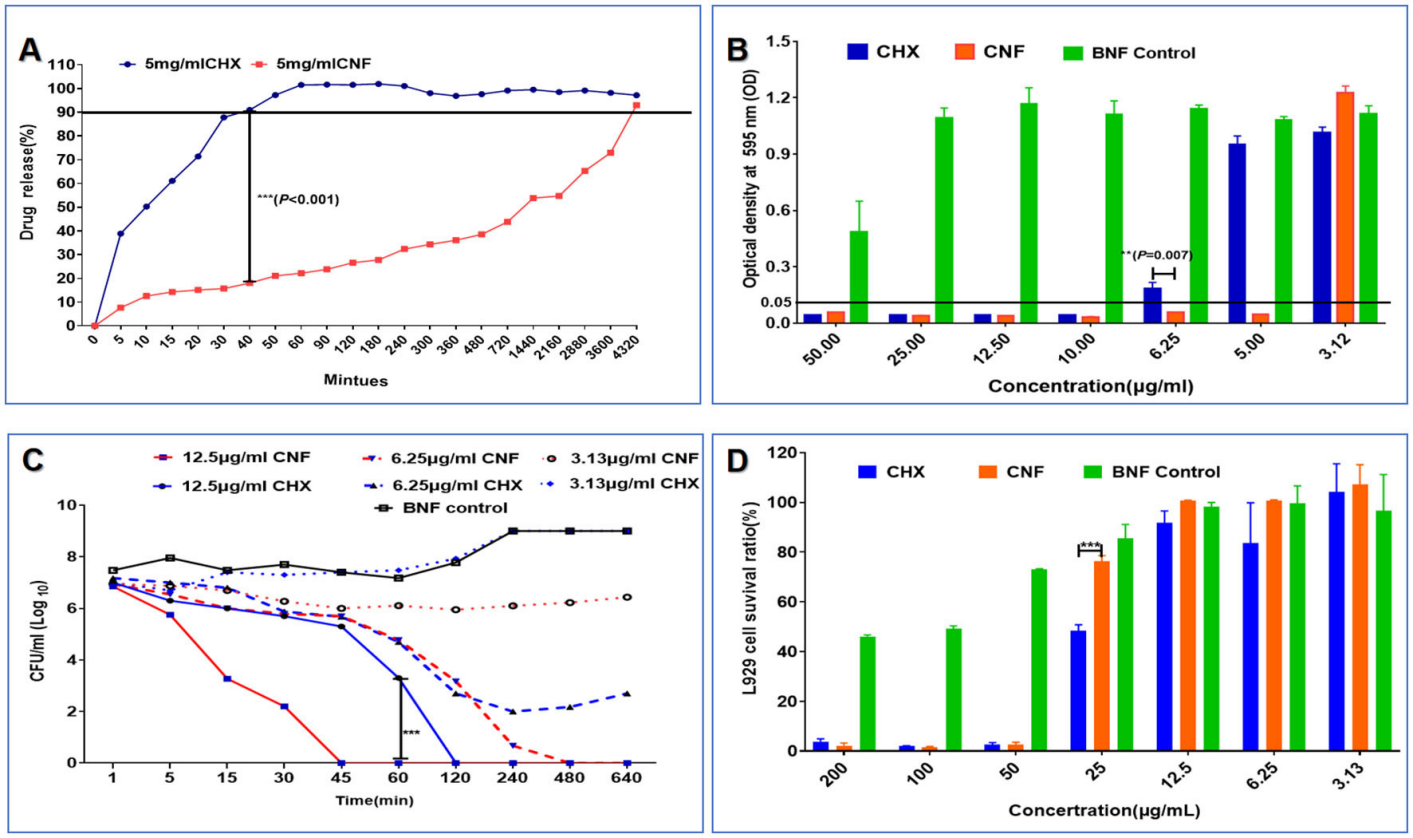
Release profile, antibacterial effect against *P. aeruginosa* and cytotoxicity of the novel nanofilm system (*n* = 3). **(A)** Release profile in SWF. **(B)** MIC. **(C)** Time–kill curve. **(D)** Cytotoxicity in L929 cells. *P < 0.05; **P < 0.01; ***P < 0.001, *T*-test.

The *in vitro* release profiles of CNF and CHX were examined in SWF release medium. Figure 5A clearly shows that CNF release was slower, whereas CHX release was exponentially faster. Significant differences between CNF and CHX were observed after 40 min (*P* < 0.001); the release rate of CHX reached 90%, whereas that of CNF was < 20% (Figure 5A). Thus, the release from CNF was significantly more retarded than that from CHX in aqueous solution.

### 3.9 *In vitro* antibacterial effects of the nanofilm system

The MIC of CNF was 5 μg/mL, the MIC of CHX was twice that of CNF, and there was no significant antimicrobial effect of BNF. There was a significant difference between the antibacterial effects of CNF and CHX at a concentration of 6.25 μg/mL (*P* < 0.01), as shown in Figure 5B. These results show that CNF exhibited stronger antibacterial effects than CHX against *P. aeruginosa in vitro*. Additionally, the bactericidal kinetics curve revealed a dose-dependent effect of drug concentration and time (Figure 5C). Compared with CHX, a concentration of 12.5 μg/mL CNF resulted in faster and more effective bactericidal activity. This novel nanofilm system, CNF, killed all *P. aeruginosa* bacteria within 45 min, whereas CHX had the same effect only at 2 h. The bactericidal kinetics of CNF showed a dose-dependent effect in terms of time and drug concentration. The addition of 6.25 μg/mL CNF resulted in a 50% reduction in bacterial viability within 2 h, and the bacteria were completely dead within ~8 h. These results show that CNF was more effective at killing *P. aeruginosa* than was CHX. Some bacteria treated with the same concentration of CHX were not killed by the end of the experiment. However, 3.13 μg/mL CHX did not significantly inhibit bacterial growth. In addition, 3.13 μg/mL CNF produced a concentration with longer-lasting inhibitory activity than did CHX. Bacterial growth was still inhibited by CNF for 16 h but was inhibited by CHX at the same concentration for only 1 h. These results show that CNF demonstrated greater antibacterial efficacy than CHX against *P. aeruginosa in vitro*.

### 3.10 *In vitro* cytotoxicity of the nanofilm system

The survival ratios of L929 cells were 3.76 ± 1.15%, 1.97 ± 0.21%, 2.59 ± 0.81%, 48.32 ± 2.42%, 91.73 ± 4.80%, 83.6 ± 16.24%, and 100.76 ± 5.66% after CHX treatment at drug concentrations of 1,000, 500, 250, 125, 62.5, 31.25, and 15.65 μg/mL, respectively, whereas after CNF treatment, the survival ratios were 2.15 ± 1.10%, 1.61 ± 0.28%, 2.53 ± 0.10%, 76.21 ± 2.39%, 100.79 ± 0.11%, 100.66 ± 0.34%, and 103.93 ± 2.90% at the same drug concentrations as CHX (Figure 5D), suggesting that this nanofilm system has no obvious cytotoxicity to L929 cells at concentrations lower than 62.5 μg/mL. Additionally, we found that the BNF control had no obvious cytotoxicity, and the survival ratios of the L929 cells treated with 125 μg/ml CNF were obviously greater than those of the L929 cells treated with CHX (*P* < 0.001). Therefore, the incorporation of CHX into the nanofilm reduced its cytotoxicity and improved its biocompatibility.

### 3.11 *In vivo* toxicity of the nanofilm system

**Figure 6:**
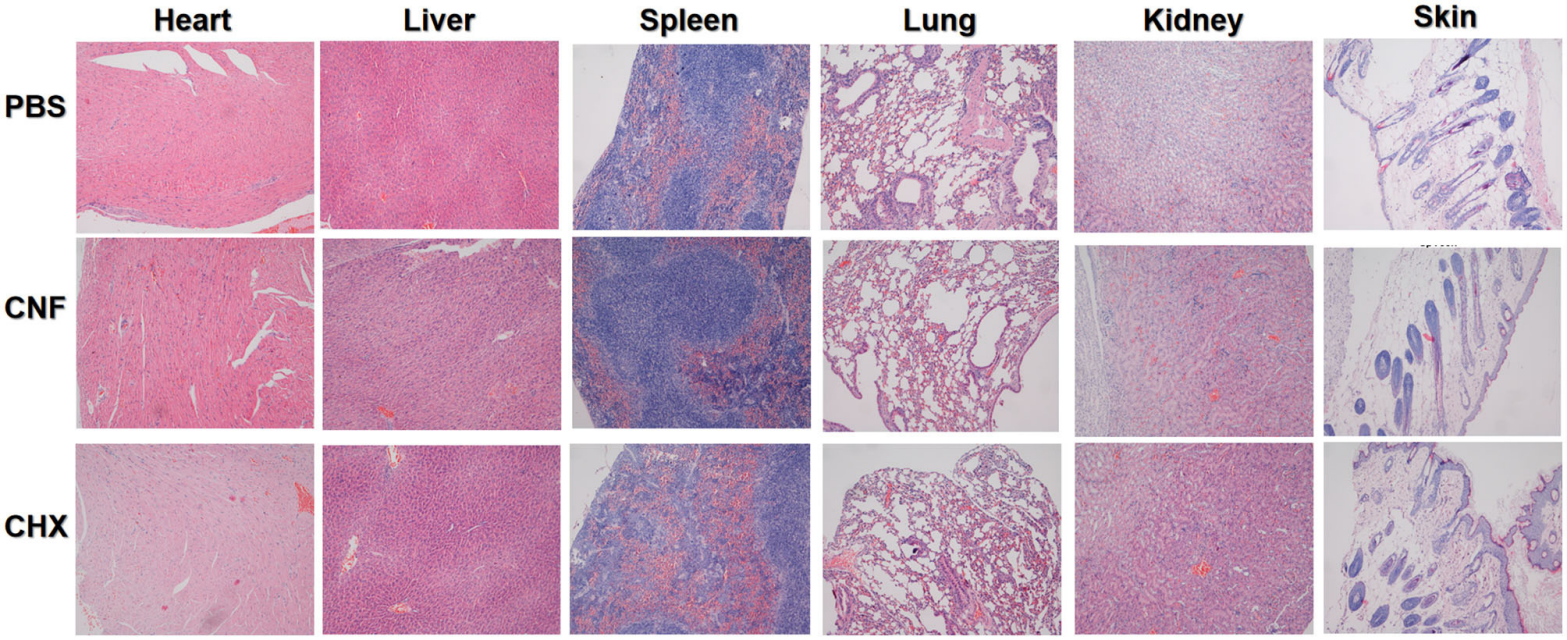
*In vivo* toxicity evaluation of the nanofilm system. Images of H&E-stained skin tissue (200 × ) after treatment for 14 days.

We further performed *in vivo* toxicity experiments to verify the toxicity in short-term experiments. The results of the examination of the tissue sections are shown in Figure 6, in which the magnification was 100 × . These results are not sufficient to confirm that CHX is only dermal toxic in the mouse wound model, and it is not clear whether there is drug accumulation in other tissues and organs. However, on the basis of these results, CNF has some protective effects owing to its nanofilm system formulation components under wound treatment conditions.

### 3.12 Changes in serum cytokine levels in the context of *P. aeruginosa* infection of wounds

**Figure 7:**
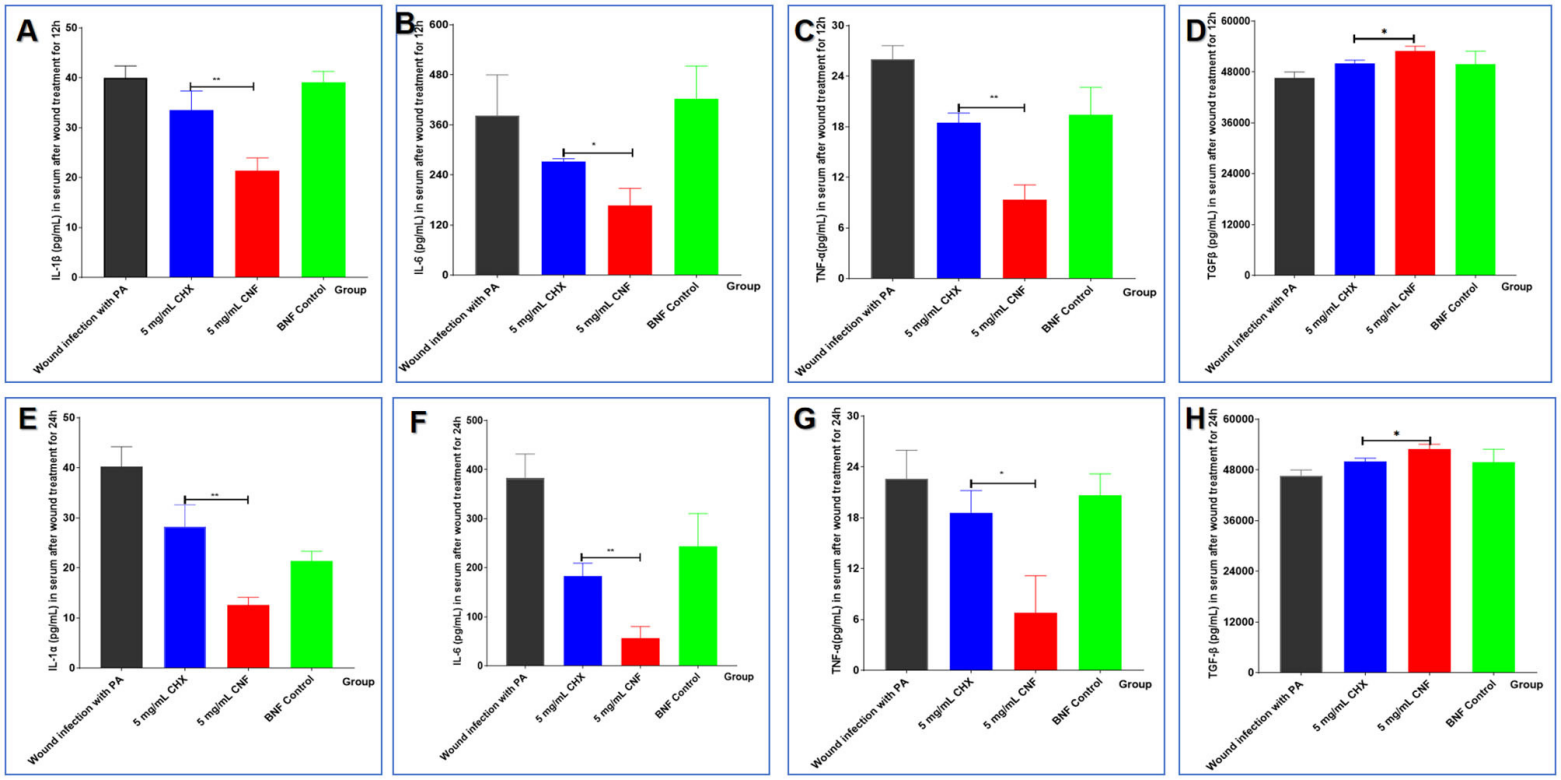
Cytokine levels in wounds infected with *P. aeruginosa*. The levels of IL-β **(A, E)**, TNF-α **(B, F)**, IL-6 **(C, G)**, and IL-10 **(D, H)** in the serum were measured 12 and 24 h after bacterial inoculation (*n* = 3). *P < 0.05; **P < 0.01; ***P < 0.001, *T*-test.

Infection with *P. aeruginosa* can not only increase the levels of the proinflammatory cytokines IL-1β, IL-6, and TNF-α but also reduce the expression of the inhibitory cytokine IL-10. These serum cytokine levels are shown in Figures 7A–H. We found that treatment with 5 mg/mL CNF significantly reduced the initial infection rate and significantly decreased the levels of cytokines (IL-β, TNF-α, and IL-6) in the host serum 12 and 24 h after infection (all *P* < 0.05). In addition, compared with those in the BNF control group, the cytokine IL-10 levels in the CNF treatment group were significantly increased and significantly greater than those in the CHX group (Figures 7D, H) (both *P* < 0.05). Therefore, CNF has a stronger anti-inflammatory effect than CHX, indicating that this dose of this nanofilm system can effectively alleviate inflammation. In summary, CNF has good bactericidal activity against skin wounds infected with *P. aeruginosa* and can thus significantly ameliorate bacterial infections and improve wound healing activity.

### 3.13 Wound healing efficacy on the basis of CFU counts

**Figure 8:**
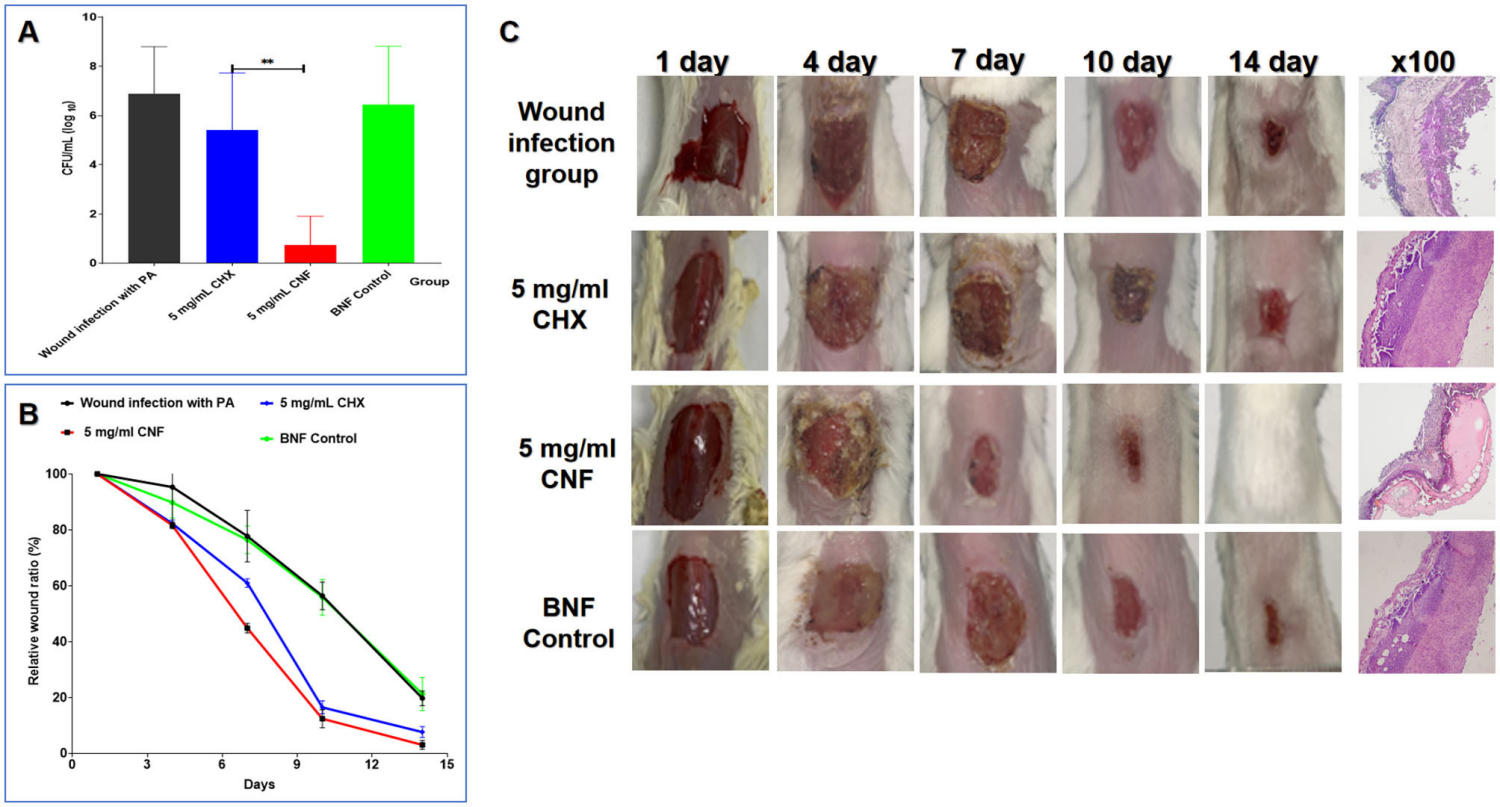
*In vivo* antimicrobial activity of the CNF in skin wounds infected with *P. aeruginosa*. **(A)** Result of bacterial colonization in the local wound (*n* = 5). **(B)** Wound healing curve (*n* = 5). **(C)** Graphical representation of the wound size changes and H&E staining results (100 × ) of the wound. Graphical representation of the results from continuous treatment for 0, 1, 3, 5, 7, and 14 days and H&E staining images of skin tissue treated continuously for 7 days. *P < 0.05; **P < 0.01; ***P < 0.001, *T*-test.

In accordance with our previous findings, CNF, CHX, and BNF were used to treat *P. aeruginosa*-infected wounds. The results showed that at the same concentration (5 mg/mL), CNF decreased the wound bacterial count faster than the aqueous solution (Figure 8A). The healing rate and healing process were evaluated to examine the ability of CNF to promote healing after wound infection. For the calculation of the healing rate, we used the mean value of the initial wound area as the denominator and the healed area of the mice as the numerator for calculating the proportion of the unhealed wound area to determine the percentage of the incision area. The results (Figure 8B) revealed that the time to initiation of scabbing was 2.66 ± 0.51 days for 5 mg/mL CNF but 4.66 ± 1.63 days for the same concentration of CHX. The time for CNF was 1.75-fold shorter than that for CHX (*P* < 0.05). The time to complete scabbing for 5 mg/mL CNF was 7.0 ± 1.41 days, which was 1.26-fold shorter than that for the aqueous solution (8.83 ± 0.75 days) (*P* < 0.05). The time to initiation of scab removal for 5 mg/mL CNF was 9.8 ± 1.09 days, which was 1.4 times shorter than that for the aqueous solution (13.8 ± 1.64 days) (*P* < 0.01). The CHX-treated group presented varying degrees of tissue erythema, and some mice also presented visible pus at 7 days.

### 3.14 General and histological examinations of wound healing

As shown in Figure 5C, after 3 and 5 days of treatment, the wound area in the CNF-treated group was smaller than that in the CHX-treated group, the BNF control group, and the PAO1 wound infection control group. We found that there was no significant difference in the general appearance of the wounds among the CHX, BNF, and infection control groups. Additionally, after 7 days of continuous treatment with 5 mg/mL CNF, the wound size was markedly reduced compared with that after CHX treatment. After continuous treatment for 14 days, complete wound healing was observed in the CNF-treated mice. These results demonstrate that CNF has good bactericidal activity against *P. aeruginosa* in damaged skin and promotes wound healing. Images of healing wounds after CNF treatment are shown in Figure 8C. Compared with CHX treatment, CNF treatment had better effects on wound healing. For example, on day 10, wound healing in the CNF group was significantly better than that in the CHX group, as the redness and thickness of the skin around the wound and scabs in the CNF group were greatly reduced compared with those in the CHX group. Histological examinations of skin wounds are important for assessing wound healing. Normal mouse skin layers are intact and clearly bound together. Once a wound is infected, inflammatory cells are highly distributed throughout the skin, and inflammatory infiltrates throughout the entire skin layer. Accordingly, as shown in Figure 8C, severe inflammation was also observed in the BNF control group. In contrast, treatment of wounds with 5 mg/mL CNF significantly reduced inflammatory infiltration in the dermis and other skin layers. In comparison, the 5 mg/mL CHX group exhibited severe inflammatory reactions and skin damage. In summary, the CNF is superior to CHX for inducing healing of skin wound infections caused by *P. aeruginosa* in mice.

## 4 Discussion

In recent years, the emergence of multidrug-resistant “superbugs” as a result of antibiotic misuse has made conventional antibiotic antimicrobial therapy much more challenging (Kwan and Beahm, 2020). Infection with multidrug-resistant *P. aeruginosa* (Rossi et al., 2021), a class I hazard, in soft-tissue injuries greatly reduces the survival rate of patients. CHX, also known as diclofenac sodium, was first developed by Imperial Chemical Industries in the UK (Poppolo Deus and Ouanounou, 2022; Seidelman et al., 2023). Owing to its broad antimicrobial spectrum and antimicrobial activity independent of body fluids, it has a relatively wide range of uses in pharmaceuticals and daily chemicals, such as mouthwash and hand sanitizers. As a cationic surfactant, its acetate solubility is low, and the aqueous solution dehydrates quickly after its application to the skin, mucous nanofilm systems and other parts of the body, which limits its application (Garcia-Contreras et al., 2019). Our previous studies (Yang et al., 2018) have shown that Benzalkonium bromide (BZL) can effectively inhibit MRSA. A novel BZL-containing liquid film-forming systems (LFFSs) were designed that is fabricated with CS and PVA and this LFFS can promote wound-healing treatment with excellent antibacterial efficacy and a delayed release effect. In the present work, a novel CNF was developed in combination with a preexisting nanoemulsion with significant antimicrobial effects and a nanofilm formulation characterized by slow release, with the expectation of obtaining a more robust and longer-lasting antimicrobial effect on wound infections with multidrug-resistant *P. aeruginosa*.

On the basis of the previously described methods nanotechnology and PVA–CS nanofilm system technology in our laboratory, we successfully prepared 5 mg/mL CNF with a ζ potential of −15.2 mV and an average size of 59.71 nm. The apparent observations, physical parameters, stability and *in vitro* release measurements revealed that the CNF had good stability, film-forming activity and slow release, addressing the limitations of CHX liquid formulations.

The *in vitro* antimicrobial results showed that the CNF has enhanced bactericidal efficiency, allowing the use of reduced concentrations of the administered drug, as well as slow release. The mechanism of action is currently unknown and needs to be further studied through investigations of the antimicrobial mechanisms of the drug, emulsion and nanofilm system formulations. We speculate that the killing effect of the nanofilm system on *P. aeruginosa* is not limited to that of the drug itself but is more likely due to the emulsion and the nanofilm system agents—surfactants and chitosan—which have a strong killing effect on the bacteria (Qin et al., 2022).

In the toxicity study of the drug administered to the wound model mice, we did not find any toxic effects beyond the skin, including major organ tissues such as the heart, liver, spleen, lungs and kidneys. According to Shanshan Zhang et al., in rats subjected to lung lavage with a high dose of CHX solution (1,000 μg/kg), drug accumulation in the lungs and kidneys results in substantial damage to those tissues. We administered a dose of 25,000 μg/kg, but because it was administered to wounded skin, toxicity was not significant, and any toxicity accumulation appeared to be more gradual once the wound had healed, which may explain the lack of toxicity. In studies of skin wound healing and changes in inflammatory factor levels, CHX has been shown to have a dose-dependent killing effect on mouse epidermal cells (Shamshad et al., 2023), whereas the application of nanofilm system agents attenuates this killing effect and inhibits the development of inflammation at the site of the wound.

However, elucidating more detailed antibacterial mechanisms against other bacteria and some problems associated with other *P. aeruginosa* infectious wound models require further study. In future studies, it may be worthwhile to explore new drug delivery methods and determine protective indicators, such as tension parameters and water correlation coefficients, after the development of nanofilm system formulations, as well as improve this carrier system or conduct antibacterial experiments with other models of infection to explore the similarities and differences among similar systems in terms of their antimicrobial potentiation effects and mechanisms (Pennington et al., 2023). The present study provides a solid basis for further research on this novel platform for CHX delivery and provides new insight for the study of drug delivery systems for liquid nanofilm system formulations.

## 5 Conclusion

In this study, we designed and developed a novel nanofilm system, CNF, with relatively high stability and high-quality characteristics, on the basis of a nanoemulsion and CS-PVA liquid film system. Additionally, this nanofilm released the incorporated drug in a sustained manner in response to the SWF. The *in vitro* results showed that this nanofilm has reduced toxicity at relatively low concentrations, and the *in vivo* results indicated low toxicity to the main organs. Importantly, we found that this novel sustained-release system not only has faster and better antibacterial effects but also better promotes healing and inhibits inflammation in a mouse model of wound infection with *P. aeruginosa*. These results suggest that this multifunctional nanofilm system with improved antibacterial effects and sustained drug release has potential for clinical application. However, further experiments are needed to explore its feasibility.

## Data Availability

The raw data supporting the conclusions of this article will be made available by the authors, without undue reservation.

## References

[B1] Arezomand Z. Mashjoor S. Makhmalzadeh B. S. Shushizadeh M. R. Khorsandi L. (2024). Citrus flavonoids-loaded chitosan derivatives-route nanofilm as drug delivery systems for cutaneous wound healing. Int. J. Biol. Macromol. 271:132670. 10.1016/j.ijbiomac.2024.132670 38806083

[B2] Cai D. Zhang Z. Feng Z. Song J. Zeng X. Tu Y.. (2022). A lipophilic chitosan-modified self-nanoemulsifying system influencing cellular membrane metabolism enhances antibacterial and anti-biofilm efficacy for multi-drug resistant *P. aeruginosa* wound infection. Biomater. Adv. 140:213029. 10.1016/j.bioadv.2022.213029 36058016

[B3] Chamchangi M. A. Abdollahi S. Raoufi Z. Badr A. A. (2024). Nano hydrogel with bacterial nanocellulose and bitter almond oil nanoemulsions for enhanced wound healing: In-vivo and in-vitro characterization. Int. J. Biol. Macromol. 277:134134. 10.1016/j.ijbiomac.2024.134134 39053828

[B4] Chen R. Wang P. Xie J. Tang Z. Fu J. Ning Y.. (2024). A multifunctional injectable, self-healing, and adhesive hydrogel-based wound dressing stimulated diabetic wound healing with combined reactive oxygen species scavenging, hyperglycemia reducing, and bacteria-killing abilities. J. Nanobiotechnol. 22:444. 10.1186/s12951-024-02687-y 39068417 PMC11283728

[B5] Chong Y. Yu D. Han R. Li Y. Gu Y. Lu Z.. (2024). Preparation of luvangetin nanoemulsions: antimicrobial mechanism and role in infected wound healing. Int. J. Nanomed. 19, 5493–5509. 10.2147/IJN.S457322 38882542 PMC11178095

[B6] Denkel L. A. Schwab F. Clausmeyer J. Behnke M. Golembus J. Wolke S.. (2022). Effect of antiseptic bathing with chlorhexidine or octenidine on central line-associated bloodstream infections in intensive care patients: a cluster-randomized controlled trial. Clin. Microbiol. Infect. 28, 825–831. 10.1016/j.cmi.2021.12.023 35031487

[B7] El-Abeid S. E. Mosa M. A. El-Tabakh M. A. M. Saleh A. M. El-Khateeb M. A. Haridy M. S. A.. (2024). Antifungal activity of copper oxide nanoparticles derived from *Zizyphus spina* leaf extract against Fusarium root rot disease in tomato plants. J. Nanobiotechnol. 22:28. 10.1186/s12951-023-02281-8 38216982 PMC10785362

[B8] Garcia-Contreras R. Wood T. K. Tomas M. (2019). Editorial: quorum network (sensing/quenching) in multidrug-resistant pathogens. Front. Cell. Infect. Microbiol. 9:80. 10.3389/fcimb.2019.00080 31001486 PMC6456689

[B9] He X. Lv Y. Lin Y. Yu H. Zhang Y. Tong Y.. (2024). Platinum nanoparticles regulated V(2)C MXene nanoplatforms with NIR-II enhanced nanozyme effect for photothermal and chemodynamic anti-infective therapy. Adv. Mater. 36:e2400366. 10.1002/adma.202400366 38469896

[B10] Hemmingsen L. M. Giordani B. Pettersen A. K. Vitali B. Basnet P. Skalko-Basnet N.. (2021). Liposomes-in-chitosan hydrogel boosts potential of chlorhexidine in biofilm eradication in vitro. Carbohyd. Polym. 262:117939. 10.1016/j.carbpol.2021.117939 33838816

[B11] Kwan A. C. F. Beahm N. P. (2020). Fosfomycin for bacterial prostatitis: a review. Int. J. Antimicrob. Agents 56:106106. 10.1016/j.ijantimicag.2020.106106 32721595

[B12] Li Y. F. Sun H. W. Gao R. Liu K. Y. Zhang H. Q. Fu Q. H.. (2015). Inhibited biofilm formation and improved antibacterial activity of a novel nanoemulsion against cariogenic Streptococcus mutans *in vitro* and *in vivo*. Int. J. Nanomed. 10, 447–462. 10.2147/IJN.S72920 25624759 PMC4296965

[B13] Luo X. Song Z. Zeng X. Ye Y. Zheng H. Cai D.. (2023). A promising self-nanoemulsifying adjuvant with plant-derived saponin D boosts immune response and exerts an anti-tumor effect. Front. Immunol. 14:1154836. 10.3389/fimmu.2023.1154836 37415983 PMC10319991

[B14] Ly T. B. Bui B. T. A. Nguyen Y. T. H. Le K. A. Tran V. T. Le P. K.. (2024). Innovative ultrasonic emulsification of cinnamon essential oil pickering emulsion stabilized by rice straw-derived cellulose nanocrystals. Int. J. Biol. Macromol. 276:134084. 10.1016/j.ijbiomac.2024.134084 39084991

[B15] Narayanan M. Alshiekheid M. A. Saravanan M. (2024). Antibacterial, mosquito larvicidal, and cytotoxicity potential of AgNPs synthesized using *Pittosporum undulatum* under *in vitro* conditions. Environ. Res. 260:119585. 10.1016/j.envres.2024.119585 39029730

[B16] Ouyang J. Bu Q. Tao N. Chen M. Liu H. Zhou J.. (2022). A facile and general method for synthesis of antibiotic-free protein-based hydrogel: wound dressing for the eradication of drug-resistant bacteria and biofilms. Bioactive Mater. 18, 446–458. 10.1016/j.bioactmat.2022.03.033 35415296 PMC8971583

[B17] Pennington E. Bell S. Hill J. E. (2023). Should video laryngoscopy or direct laryngoscopy be used for adults undergoing endotracheal intubation in the pre-hospital setting? A critical appraisal of a systematic review. J. Param. Pract. 15, 255–259. 10.1002/14651858 PMC7616025 38812899

[B18] Poppolo Deus F. Ouanounou A. (2022). Chlorhexidine in dentistry: pharmacology, uses, and adverse effects. Int. Dental J. 72, 269–277. 10.1016/j.identj.2022.01.005 35287956 PMC9275362

[B19] Qin S. Xiao W. Zhou C. Pu Q. Deng X. Lan L.. (2022). *P. aeruginosa*: pathogenesis, virulence factors, antibiotic resistance, interaction with host, technology advances and emerging therapeutics. Signal Transd. Targ. Ther. 7:199. 10.1038/s41392-022-01056-1 35752612 PMC9233671

[B20] Ramirez-Labrada A. Santiago L. Pesini C. Arrieta M. Arias M. Calvo Perez A.. (2024). Multiparametric *in vitro* and *in vivo* analysis of the safety profile of self-assembling peptides. Sci. Rep. 14:4395. 10.1038/s41598-024-54051-7 38388659 PMC10883997

[B21] Rezaie M. Rafiee Z. Choi S. (2024). Unlocking wearable microbial fuel cells for advanced wound infection treatment. ACS Appl. Mater. Interf. 16, 36117–36130. 10.1021/acsami.4c06303 38950522

[B22] Rossi E. La Rosa R. Bartell J. A. Marvig R. L. Haagensen J. A. J. Sommer L. M.. (2021). *P. aeruginosa* adaptation and evolution in patients with cystic fibrosis. Nat. Rev. Microbiol. 19, 331–342. 10.1038/s41579-020-00477-5 33214718

[B23] Salih E. Mgbeahuruike E. E. Prevost-Monteiro S. Sipari N. Vare H. Novak B.. (2024). Polyphenols and phenolic glucosides in antibacterial twig extracts of naturally occurring *Salix myrsinifolia* (Salisb.), *S. phylicifolia* (L.) and S. starkeana (Willd.) and the cultivated hybrid S. x pendulina (Wender.). Pharmaceutics. 16:16070916. 10.3390/pharmaceutics16070916 39065613 PMC11280161

[B24] Sang Y. Gao J. Han X. Liang T. Chen T. Zhao Y.. (2024). Preparation and sustained release of diatomite incorporated and Eudragit L100 coated hydroxypropyl cellulose/chitosan aerogel microspheres. Int. J. Biol. Macromol. 267:131447. 10.1016/j.ijbiomac.2024.131447 38588843

[B25] Seidelman J. L. Mantyh C. R. Anderson D. J. (2023). Surgical site infection prevention: a review. JAMA 329, 244–252. 10.1001/jama.2022.24075 36648463

[B26] Shamshad F. Khan S. Zamir S. W. Khan M. H. Hayat M. Khan F. S.. (2023). Transformers in medical imaging: a survey. Med. Image Anal. 88:102802. 10.1016/j.media.2023.102802 37315483

[B27] Soleymani F. Rahimi H. R. Farsiani H. Jalili A. (2024). Antimicrobial activity of chitosan scaffold loaded with soluble factors of different probiotic strains against multidrug resistant *P. aeruginosa*. Iran. J. Biotechnol. 22:e3612. 10.30498/ijb.2024.381455.3612 38827340 PMC11139447

[B28] Valappil S. P. Abou Neel E. A. Zakir Hossain K. M. Paul W. Cherukaraveedu D. Wade B.. (2024). Novel lactoferrin-conjugated gallium complex to treat *P*. aeruginosa wound infection. Int. J. Biol. Macromol. 258:128838. 10.1016/j.ijbiomac.2023.128838 38128798

[B29] Wang K. Liu Y. Wang H. Liu Y. Yang X. Sun S.. (2022). Multi-functional nanofilms capable of angiogenesis, near-infrared-triggered anti-bacterial activity and inflammatory regulation for infected wound healing. Biomater. Adv. 142:213154. 10.1016/j.bioadv.2022.213154 36341743

[B30] Wen M. M. Abdelwahab I. A. Aly R. G. El-Zahaby S. A. (2021). Nanophyto-gel against multi-drug resistant *P. aeruginosa* burn wound infection. Drug Deliv. 28, 463–477. 10.1080/10717544.2021.1889720 33620004 PMC7906617

[B31] Xie T. Q. Yan X. Yan J. H. Yu Y. J. Liu X. H. Feng J.. (2024). Construction of iron-scavenging hydrogel via thiol-ene click chemistry for antibiotic-free treatment of bacterial wound infection. Adv. Healthcare Mater. 13:e2401118. 10.1002/adhm.202401118 38979865

[B32] Yang S. Yang Y. Cui S. Feng Z. Du Y. Song Z.. (2018). Chitosan-polyvinyl alcohol nanoscale liquid film-forming system facilitates MRSA-infected wound healing by enhancing antibacterial and antibiofilm properties. Int. J. Nanomed. 13, 4987–5002. 10.2147/IJN.S161680 30214202 PMC6128272

[B33] Yassin A. Albekairy A. Omer M. Almutairi A. Alotaibi Y. Althuwaini S.. (2023). Chitosan-coated azithromycin/ciprofloxacin-loaded polycaprolactone nanoparticles: a characterization and potency study. Nanotechnol. Sci. Appl. 16, 59–72. 10.2147/NSA.S438484 38146545 PMC10749578

[B34] Zhou J. Ji X. Wang H. Hsu J. C. Hua C. Yang X.. (2024). Design of ultrasound-driven charge interference therapy for wound infection. Nano Lett. 24, 7868–7878. 10.1021/acs.nanolett.4c00930 38912706 PMC11334693

[B35] Zulfiqar S. Sharif S. Nawaz M. S. Shahzad S. A. Bashir M. M. Iqbal T.. (2024). Cu-MOF loaded chitosan based freeze-dried highly porous dressings with anti-biofilm and pro-angiogenic activities accelerated *P. aeruginosa* infected wounds healing in rats. Int. J. Biol. Macromol. 271:132443. 10.1016/j.ijbiomac.2024.132443 38761913

